# Making Sense of Non-Response: What Patients Teach Us About Concentrated Exposure Therapy for Obsessive Compulsive Disorder

**DOI:** 10.3390/jcm15156032

**Published:** 2026-08-03

**Authors:** Lara Wille, Luisa Tegtmeier, Amir H. Yassari, Jakob Scheunemann, Silke Pawils, Franziska Miegel, Lena Jelinek

**Affiliations:** 1 Department of Psychiatry and Psychotherapy, University Medical Center Hamburg-Eppendorf, Martinistrasse 52, 20246 Hamburg, Germany; la.wille@uke.de (L.W.); l.tegtmeier@uke.de (L.T.); a.yassari@uke.de (A.H.Y.); j.scheunemann@uke.de (J.S.); l.jelinek@uke.de (L.J.); 2 Department of Medical Psychology, University Medical Center Hamburg-Eppendorf, Martinistrasse 52, 20246 Hamburg, Germany; s.pawils@uke.de

**Keywords:** intention-focused, OCD, ERP, B4DT, non-response

## Abstract

**Background/Objectives:** The Bergen 4-Day Treatment (B4DT) is a concentrated form of exposure and response prevention for patients with obsessive–compulsive disorder (OCD). Although response rates for the B4DT are generally high, a proportion of patients does not achieve clinically significant improvements. Quantitative measures can identify non-response, but offer limited insight into processes that may hinder change in highly structured, time-limited interventions. To identify factors potentially related to the lack of improvement, this study explored experiences of patients who had not benefitted from B4DT. **Methods:** B4DT participants were recruited from an uncontrolled German study. Of 32 patients assessed at three-month follow-up, 12 met the non-response criteria (<35% reduction on the Yale–Brown Obsessive Compulsive Scale), and eight completed semi-structured qualitative interviews 9–14 months after treatment. Interviews were analyzed using grounded theory methods. A brief quantitative comparison examined baseline differences between responders and non-responders. **Results:** Participants described benefits from B4DT, including improved understanding of OCD, supportive peer contact, and increased courage for exposure tasks. However, they consistently reported difficulties sustaining self-guided exposure after treatment. Within the B4DT framework, one possible interpretation is that the intended shift toward independently approaching anxiety had not yet been fully consolidated. **Conclusions:** Although quantitatively classified as non-responders, participants described meaningful psychological benefits from B4DT. The findings suggest that non-response may reflect incomplete consolidation of the intended shift from symptom control toward approaching anxiety in service of personally meaningful action, rather than an absence of change.

## 1. Introduction

Exposure and response prevention (ERP) is considered the first-line, gold standard treatment for obsessive–compulsive disorder (OCD) [1,2]. Despite this, a significant number of patients do not respond to ERP [3,4,5]. Recently, research interest in concentrated exposure therapy (cET) has grown, with particular attention to the Bergen 4-Day Treatment (B4DT [6]). This approach has demonstrated high response and remission rates, but not all patients respond to B4DT [6]. Response to treatment is defined differently across disorders [7,8,9,10]. For OCD, a common definition of treatment response is a decline in symptoms of at least 35% on the Yale-Brown Obsessive Compulsive Scale (Y-BOCS [11]).

In B4DT, individuals diagnosed with OCD receive a personalized, exposure-based treatment over four consecutive days (approximately 25 therapy sessions in total). The treatment is provided by a multidisciplinary team of psychiatrists, psychologists, and nurses and combines individual and group psychotherapy. B4DT has proven to be highly effective in uncontrolled studies [6,12,13], one randomized controlled trial comparing B4DT with self-help and a waiting-list control [14], and even among individuals who had previously failed to respond to conventional ERP [15], highlighting its potential as an effective intervention in otherwise treatment-resistant OCD. Results show high and lasting improvement rates, with response rates between 80% and 95% and remission rates of 56–75% over months to years [6,12,14,15,16,17]. Thus, participants have experienced significant improvement in a much shorter timeframe compared to regular therapeutic approaches. More recently, an uncontrolled study on B4DT in Germany—the dataset on which the present paper is based—reported a response rate of 63% [18]. Hence, in this and other studies, 10–37% of participants were classified as non-responders at follow-up assessment. Thus, despite its strengths, a meaningful non-responder proportion also appears in B4DT trials. Although quantitative outcome measures can assess who benefits from treatment, they offer little insight into the subjective processes and barriers that may hinder clinical improvement.

Qualitative approaches are particularly well suited to address this gap as they allow for an in-depth exploration of patients’ subjective experience of treatment, including perceived facilitators, barriers, and moments of stagnation [19]. Qualitative research underscores the importance of relational, contextual, and experiential factors for recovery [20,21]. In cET, factors such as motivation, readiness for change, and courage may be particularly relevant as they can shape how patients engage with demanding exposure tasks [22]. However, in B4DT, which can be described as an intention-focused treatment, the goal is not to simply push through feared situations but to intentionally approach anxiety with openness, ownership, and active engagement [22,23,24]. Using the “Leaning into Anxiety” (LET) protocol, patients change their intention from avoiding uncomfortable feelings such as fear to embracing them.

Existing research remains insufficiently specific regarding the processes that may distinguish responders from non-responders in highly structured, time-limited exposure treatments such as B4DT. The present study therefore aimed to explore the treatment experiences of patients who did not respond sufficiently to the B4DT. Through qualitative interviews with non-responders, we sought to identify factors that initiated engagement, as well as elements that may have hindered the transfer of therapeutic gains into everyday life. By examining these experiences, this study may contribute to a more differentiated understanding of non-response in concentrated exposure-based treatments—in this case, the B4DT—and inform potential refinements of the treatment.

## 2. Materials and Methods

### 2.1. Participants

B4DT non-responders were recruited from an uncontrolled study on B4DT in Hamburg, Germany. For a detailed description, see Jelinek et al. 2024 [25]. In this study, the final total sample consisted of 33 individuals diagnosed with OCD according to the MINI International Neuropsychiatric Interview (MINI [26]). Participants were assessed with the Y-BOCS before B4DT (T0), two weeks after B4DT (T1), and at follow-up three months after treatment (T2). One patient did not participate in the T2 assessment (lost to follow-up); thus, data from *N* = 32 participants were available, with *n* = 20 (62.5%) classifying as responders (defined according to Mataix-Cols et al. (2016) [11] as ≥35% reduction in the Y-BOCS between T0 and T2) and *n* = 12 (37.5%) classified as non-responders at follow up assessment (<35% decline on the Y-BOCS between T0 and T2). We re-contacted non-responders to interview them for the present study. One person did not agree to participate, and three people could not be reached, leaving a final sample of *n* = 8 non-responders for the qualitative analysis, and a sample of *n* = 12 for the descriptive comparison.

### 2.2. General Procedure

The interviews took place nine to 14 months after the treatment (from March to July 2024). A semi-structured interview was developed for the qualitative interviews (see Appendix A). The participants were told that the aim of the interview was to assess their personal experience with B4DT: what was helpful, but especially what was missing and what could be improved.

The semi-structured interview consisted of 10 open-ended questions and covered the following themes: (a) subjective changes in behavior and emotions resulting from the treatment and their evaluation and attribution, (b) subjective reasons for lack of improvement after B4DT, (c) participants’ subjective resources as well as factors that potentially held them back throughout the B4DT, (d) participants’ feedback on the B4DT process, and (e) suggestions for improving B4DT.

The interviews were conducted by a psychologist in postgraduate training for cognitive behavior therapy (LT). Five interviews were conducted in person, while three interviews were conducted via video conference for logistical reasons. No notable differences were observed in engagement between the two formats. The interviews lasted between 35 and 90 min. The conversations were recorded and transcribed verbatim.

The project was approved by the Local Psychological Ethics Committee at the Center for Psychosocial Medicine (LPEK-0730). All participants received written and verbal information about the study, including details regarding the audio recording of the interview, data security, voluntary participation, and the possibility of withdrawing from the study at any time without negative consequences. Any names mentioned during the interview were anonymized during transcription, and the audio files were deleted after transcription. No financial compensation was offered for participation. All participants gave written informed consent.

### 2.3. Analysis

In qualitative research, sample size is determined by way of saturation, the point at which new cases do not add new themes [27]. Saturation is often achieved between nine and 17 participants [28]. For the present study, sample size was determined by the limited number of eligible non-responders available for recruitment.

For the analysis of the qualitative data of the eight non-responders that agreed to be interviewed, we applied an inductive approach based on constructivist grounded theory [29] to extract recurring patterns and central themes from the participants’ statements. The software MAXQDA 2023 was used for this purpose (https://www.maxqda.com, accessed on 1 August 2026). We divided the analysis process into three phases, which were conducted iteratively and partially in parallel.

After transcription, summary reports were created for each case to capture the core content. In the initial coding phase, the interviews were subjected to open coding. The texts were systematically processed by identifying relevant passages and assigning descriptive codes while staying close to the data [30]. These codes were continuously compared within and across transcripts to define commonalities between the experiences of the non-responders.

In the second phase, focused coding, we evaluated the initial codes regarding their relevance, frequency, and explanatory power [30]. Codes that captured particularly salient aspects of the participants’ experience were selected and applied to larger segments of the data. This step allowed for reduction and consolidation of the coding system. Finally, in theoretical coding, we organized the codes into a coherent structure by examining relationships between categories and subcategories, thereby clarifying how these categories were connected within the process experienced by non-responders [30].

Two psychologists independently conducted the coding of the transcripts and regularly conferred to refine the definition of categories. Throughout the entire process, memos were used to document and exchange thoughts, ideas, and hypotheses, including suggestions for alternative systems of subcategories. Reflexive memos documenting assumptions, potential biases, and alternative interpretations were maintained throughout the analysis. Further, themes were cross-checked with other OCD researchers and B4DT therapists as part of a peer debriefing process. Discrepancies were resolved through discussion.

We included quantitative analysis to assess baseline differences between responders and non-responders, as well as between non-interviewed and interviewed non-responders, using Welch’s *t*-tests [31], Fisher’s Exact Test, and the Fisher-Freeman-Halton exact test [32] as appropriate. To descriptively characterize non-responders’ compliance during treatment, therapist ratings from the exposure days were included as raw data.

## 3. Results

### 3.1. Quantitative Results

**Figure 1:**
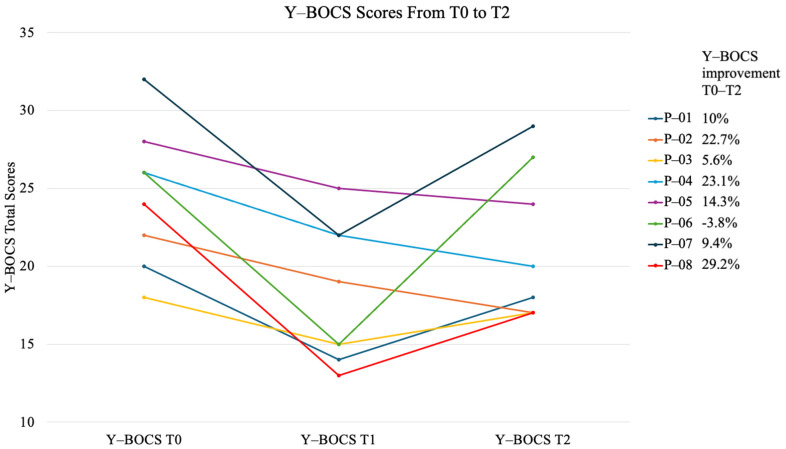
Y-BOCS Scores From T0 to T2 for the Eight Interviewed Non-responders. Note. Y-BOCS = Yale-Brown Obsessive Compulsive Scale, total score. T0 refers to baseline, T1 refers to post-treatment, and T2 refers to the follow-up three months after treatment. Y-BOCS improvements from T0 to T2 are presented as percentages for each participant.

The final sample included five women (62.5%) and three men (37.5%), with a median age of 41 years (range 26 to 67). For more details on demographics, see Table 1. Responders and non-responders did not differ significantly regarding gender, relationship status, occupation, illness duration, age of onset, medication, or comorbidities. However, non-responders were significantly older than responders (Table 1). Regarding their symptom improvement, the Y-BOCS scores indicate that non-responders numerically improved over the course of the treatment days (T0 to T1) (Figure 1; Table 2). While three participants improved slightly more from after the treatment to follow-up at T2, the remaining five non-responders experienced an increase in OCD symptoms during this period (Figure 1). With respect to participants’ engagement and implementation of the B4DT technique throughout the four treatment days, therapists assigned high ratings to the non-responders regarding their completion of planned exposures, spontaneous practice, application of the LET technique, and overall effort (Table 3). We observed no meaningful differences between interviewed and non-interviewed participants, although these comparisons were severely underpowered and should therefore be interpreted cautiously.

The following sections present the themes derived from the qualitative interviews with the eight non-responders. An overview of all final categories including descriptions and quotes are displayed in Table 4.

### 3.2. Qualitative Results

Consistent with the results in the quantitative analysis, participants described various positive developments throughout their treatment process. They reported entering the treatment with hope and motivation, gaining a deeper understanding of their disorder, developing the courage to engage challenging situations, and experiencing support from their treatment group.

#### 3.2.1. Initial Engagement

##### Motivation and High Hopes

Most participants described an increase in motivation when they decided to sign up for the B4DT and during the preparation phase prior to the four treatment days. Importantly, participants seemed to use the term “motivation” to describe a sense of hope and positive expectations regarding the possibility of symptom improvement rather than motivation to take on demanding therapeutic work. Especially if they had failed previous treatment, hearing about the success rate of B4DT seemed to instill a sense of hope. Other participants also mentioned their family members as a reason for entering the treatment, suggesting that their motivation was also connected to a wish to reduce the burden on their loved ones.

“*I felt more motivated to tackle my OCD. I felt motivated as soon as I heard about the therapy. My daughter was six months old at the time. Next to my husband, she was the biggest motivation to get rid of the whole thing.*”

[P-01]

“*I have experiences with therapy because I have had this disorder for more than 50 years and I was looking for something new and I found this, so I signed up in the hope that it might help.*”

[P-52]

“*Before the week started, my expectation was that my OCD would improve significantly. And I have to say I entered the week fully motivated. But I did not expect it would be gone completely.*”

[P-48]

#### 3.2.2. Positive Change During Treatment

Throughout the four treatment days, participants described a process of change within the structured and supportive treatment context. They gained a new understanding of their disorder, and successfully conducting exposures evoked a feeling of courage.

##### Understanding the Disorder and the Treatment

Overall, participants described that the treatment helped them to gain a better understanding of their disorder and of their obsessions and compulsions and how their behavior was reinforcing their OCD. The psychoeducation included the rationale for ERP and the specific techniques used in B4DT. Participants reported that this laid the foundation for the rest of the treatment, preparing them for the subsequent exposure exercises.

“*Receiving such a detailed explanation of the theoretical background was important and helpful.*”

[P-07]

“*The explanation at the beginning helped me, even though I didn’t immediately internalize it.*”

[P-04]

“*Basic knowledge/scientific perspective—understanding what is going on inside you.*”

[P-02]

Participants emphasized that this understanding did not translate into immediate mastery but still created a sense of clarity that supported their willingness to proceed with challenging therapeutic exercises.

##### Courage Through Exposure

Participants’ motivation or hope combined with their new understanding of their disorder and its treatment apparently allowed them to engage in the next step in their treatment process: exposure exercises. The lived experience of confronting their fears fostered courage, which helped them muster the strength necessary for the treatment days. Although this courage did not seem sufficient to translate the learned techniques into their everyday life, it was still reported as a relevant part of the treatment process.

“*Even though my compulsions got worse again afterwards, the week gave me a lot of courage, and whenever I think back on it, it gives me courage because I can put myself back in that feeling.*”

[P-06]

“*I have become more courageous, exposing myself to situations that I would otherwise never have considered. I have learned that difficult situations are bearable and not as bad as I thought they would be.*”

[P-03]

At the same time, participants noted that this courage was closely tied to the treatment context and did not consistently persist once the external structure and guidance were removed.

##### Peer Support from Fellow Participants

The group-based format of the B4DT program was consistently described as a beneficial component of the intervention. The opportunity for exchange with other individuals affected by OCD was experienced as validating, reduced feelings of isolation, and further helped patients feel motivated and courageous.

“*Getting through the week as a group and sharing experiences with others really helped me a lot.*”

[P-06]

#### 3.2.3. The Transition to Independent Practice

Despite the progress the participants described during their concentrated treatment days, a recurring process emerged around the transition from the concentrated treatment days to independent practice. Participants experienced this progression as abrupt and demanding, and many described a sense of losing momentum once the external structure and support of the program were removed. Across accounts, this “handover” problem from supported to self-guided action was identified as a critical point at which change became difficult to sustain.

##### Desire for Ongoing Support

The main reason given by participants for the lack of success of the treatment was the lack of therapeutic guidance after the four treatment days. All participants found it challenging to implement ERP into their everyday life after the four-day treatment ended. In particular, the implementation of the 30-day plan—which involved completing self-guided exposure exercises in the month following the four-day therapy—was perceived as difficult. Some managed to apply the learned techniques for weeks on their own, but eventually all participants reverted to their previous behavioral patterns. Most participants explicitly stated a desire for continued guidance and support after the four days of therapy.

“*And then we got the (30-day) plan (for after the four treatment days), and I pretty much failed right away—I wished for and needed someone who could help me and knew what they were doing. It was too fast.*”

[P-04]

“*I would say that for about a month after the treatment, it was noticeably better, and then somehow it totally collapsed, and I somehow couldn’t manage to maintain everything I had learned during that week.*”

[P-06]

“*For me, it would have been important to have some aftercare, a more intensive support afterwards, for example once a month or once a quarter.*”

[P-08]

“*A lot happened during those four days, and I needed some rest at first, but then I would have wished for a refresher after about two weeks, maybe for one or two days with guided exposure.*”

[P-03]

“*During the four days, it was very motivating, but I noticed that afterwards I missed having someone by my side. I know it’s not the purpose of concentrated therapy, but it would be helpful to have follow-up treatment. Maybe weekly at first and then less frequently. But to agree on goals together and then have a fixed day to discuss them.*”

[P-01]

“*I couldn’t talk to anyone after the treatment, and it’s also very short. For me, it would have been nicer if it had been longer.*”

[P-02]

“*So, in my opinion, there was no proper follow-up care.*”

[P-07]

Participants described the contrast between the highly structured treatment days and the autonomy required to sustain change by themselves after the four days of the intervention as particularly difficult, highlighting how support functioned as a stabilizing element during change.

##### Safety Behavior During Exposure

Discussing the role of therapeutic support, participants described the therapist’s presence as providing safety, reassurance, or external permission during exposure. Some experienced guided ERP exercises very positively; they specified aspects such as receiving reassurance and not feeling afraid because they were not alone.

From a learning-based perspective, this could indicate that therapist presence functioned as a safety cue for some participants.

[Interviewer: “*Were there any aspects of the concentrated treatment that were difficult or painful, but nevertheless acceptable or perhaps even helpful?*”] Participant: “*I can’t think of any. Because of the therapeutic support, I felt good. The fact that someone said, “You are allowed to do this now,”* i.e.*, this confirmation from the outside.*”

[P-04]

“*I didn’t (feel afraid). The therapist actually gave me safety, so it wasn‘t actually painful.*”

[P-05]

This reliance on external reassurance appeared to shape how participants experienced exposure, which may have affected their ability to tolerate distress without support after treatment.

##### Treatment Time

Many participants stated they would have liked to have more time to practice ERP before continuing completely by themselves. One person stated that they needed more time because it took them a while to adjust to the social setting. Most participants stated that one or two more days of guided exposures would have been important to feel confident enough to continue using the learned techniques after the program.

“*Because it started right away in the group, and I think it took me a day or two to really open up about everything.*”

[P-06]

“*You can’t do that in such a short time. I didn’t have enough time; maybe three more days would have been enough.*”

[P-05]

“*And even more time for the exposures; in fact, we only did two days.*”

[P-07]

“*I would perhaps extend the training phase by one day so that you can practice more, and then maybe after a week you could have another task and discuss it, and then slowly phase it out.*”

[P-04]

These accounts suggest that the pace of the transition from guided to independent exposure was experienced as too rapid for sustained integration of the techniques.

##### Symptom Recognition

Some patients described difficulties that arose because they gained understanding regarding their symptomology during the program. They felt like they only fully grasped the content of their OCD at the end of the four treatment days and had insufficient opportunity to practice how to navigate symptoms they had only identified within the treatment process. This also led to the feeling of not being the “right” target group.

“*So, I couldn’t really expose myself to my thoughts. I thought exposures weren’t that important for me because I ruminate constantly anyway. That’s why I didn’t feel like I was the right target group. I once said that I find it difficult to leave the apartment when a candle was lit beforehand, and then they fixated on that, but that wasn’t my main problem.*”

[P-02]

“*I think that the treatment really focuses on these exposures, and it definitely helped to practice so intensively, but I think that in my case, where there is so much going on in my head, I don’t know if you can do such extreme exposures. I think it was missing a bit more of a focus on the fact that it’s really in my head, but I only became aware of everything in the course of the week.*”

[P-06]

Thus, for some participants one difficulty was a shift in their understanding of the disorder during the treatment; by the time they understood their symptoms within the treatment model and were able to select suitable exposures, the treatment was already in its final stages.

##### Amplifiers

Some participants identified additional factors that hindered the handover process. These factors appeared as amplifiers rather than primary causes of non-response. Comorbid depressive symptoms reduced energy for applying ERP once symptoms amplified, while personal characteristics such as low self-esteem, perfectionism, and limited stress tolerance made sustained change feel risky and overwhelming. For one patient, a challenging diagnostic process contributed to lingering doubts about the diagnosis itself. This patient received an OCD diagnosis in the psychiatric outpatient center only after a lengthy diagnostic process due to some uncertainty on the clinician’s side. The patient was referred to the B4DT; however, this process left the patient feeling unprepared and without a sense of having actively decided to participate in the program. Finally, lack of social support or ongoing relational strain drained energy and interfered with continued practice.

“*After about a month, I was no longer able to maintain what I had learned during the week—the depressive symptoms came back and made everything harder.*”

[P-06]

“*This idea that I mustn’t do anything wrong, this absolute perfectionism. That made it difficult for me.*”

[P-04]

“*My relationship at the time—I did not receive any support. It actually drained my energy because I was trying to keep the relationship going.*”

[P-03]

Across interviews, a consistent pattern emerged: Although participants experienced the process as meaningful under conditions of intensive support, they struggled to sustain changes once the responsibility for the exposure exercises was shifted fully to them.

## 4. Discussion

This study explored how participants defined as non-responders to the Bergen 4-Day Treatment (B4DT) experienced the treatment process. In sum, participants reported positive changes throughout the treatment, but consistently emphasized difficulties transferring and sustaining these changes in everyday life once the external structure and therapist support were removed.

Overall, participants experienced positive change throughout the B4DT. First, they gained a better understanding of their disorder from the psychoeducation provided in the treatment. This aligns with quantitative data from the same pilot study, indicating an increase in disorder-related insight during the treatment (based on data collected before and after each treatment day; see Miegel et al. 2025 [33]). Second, participants learned how to plan and execute exposure exercises. Therapist ratings indicate that the interviewed non-responders were generally able to complete planned exposures and showed high levels of effort. Additionally, symptom severity improved numerically for all non-responders by the end of the B4DT treatment days.

However, participants described the period immediately following treatment as destabilizing and overwhelming, largely due to the “sudden” absence of structured therapeutic support. This gap between in-session engagement and post-treatment autonomy emerged as a potentially important theme in participants’ accounts of non-response. This difficulty aligns with previous findings showing symptom rebounds are common during this early post-treatment period of ERP [34]. Thus, the shift from intensive therapeutic structure to complete autonomy may present a vulnerable period in which initially acquired skills are not consistently sustained.

Why was the transition to post-treatment so challenging for non-responders? We interpret these accounts as reflecting an incomplete shift in intention. The most prominent theme across participants’ accounts was the difficulty moving from therapist-supported treatment to independent practice post-treatment. The treatment structure, the group support, and therapeutic guidance appeared to facilitate their engagement, and applying the learned technique seemed difficult once these factors were removed. One possible interpretation, informed by the B4DT model, is that the intended shift from symptom-control efforts toward independently approaching anxiety had not been fully achieved. Without the adoption of an intention-focused stance—characterized by choosing anxiety rather than pushing through it—exposure may remain effortful, compliance-based, and context-bound, thereby limiting deeper learning, generalization, and long-term symptom change. Participants described entering B4DT with hope, positive expectations, and self-described motivation. However, expectations and readiness for treatment participation may not equate to a sustained commitment to engaging in demanding changes in intention and behavior. Although positive expectations appear to have positive effects on therapy engagement [33,35], other research on B4DT has found that motivation does not significantly predict symptom improvement [36]. In relation to our findings, this may suggest that hope and motivation facilitate engagement but may be insufficient to produce therapeutic change unless accompanied by active execution of treatment principles.

Further, participants also reported experiencing an increase in courage throughout the treatment, especially due to conducting ERP. However, courage or bravery may not be sufficient to support long-term changes in B4DT; sustained change requires a shift in intention to approach and embrace anxiety instead of pushing through a supposedly dangerous situation [37,38]. While courage may enable participation within a highly structured treatment context, it does not necessarily lead to continued, self-guided engagement once external scaffolding is removed.

As a related process, some participants reported feeling little or no anxiety or distress during ERP. One possible interpretation is that, for these participants, the therapist’s presence functioned as a safety signal, reassuring them that nothing bad would happen. In that scenario, this may have limited the opportunities for participants to experience themselves as independently capable of engaging with feared situations. Guided ERP is a crucial step in treatment as it helps patients apply the LET technique, engage in ERP, and remain present with their thoughts and feelings. At the same time, intensive therapist involvement may foster passivity if patients become too reliant on external guidance. This reliance can be understood as a form of safety behavior [39]. In line with this interpretation, research indicates that while the number of guided exposure sessions predicts treatment outcome, self-guided exposure appears to be particularly associated with an increase in self-efficacy [2]. This pattern offers a possible explanation of why participants who relied on the therapist’s presence showed limited symptom improvement; they may not have experienced themselves as independent agents in their therapeutic process. Thus, paradoxically, therapeutic guidance—which is believed to be essential for treatment success—may inadvertently limit the autonomy that is also needed for lasting change.

Beyond difficulties in the transition from intensive therapist-supported treatment to independent practice, participants described individual, contextual, and treatment-related factors that may have contributed to non-response. For one participant, depressive symptoms appeared to reduce the energy and persistence needed to continue independent exposure practice. While some literature states that severe depressive symptoms can predict worse outcomes in OCD treatment, research on the B4DT did not find a significant relationship between pre-treatment depressive symptoms and treatment outcome [40]. Still, in this particular case, the depressive symptoms returned after OCD treatment had been completed, and it seems clinically relevant to monitor such developments and possibly offer booster sessions to maintain achieved changes. A lack of social support may also have contributed to the challenges in transitioning from treatment to everyday life. Studies support the notion that social support plays a relevant role in OCD treatment, for example by affecting levels of readiness to change [41,42]. Some participants may have also been affected by occupational stress—while there is not much evidence specifically for stress in the workplace and OCD, stress in general can be both a trigger and a maintaining factor for OCD symptoms [43]. Further, a perfectionistic approach to independent ERP was also described as a challenge. From a clinical perspective, it is plausible that higher levels of perfectionism may interfere with self-guided exposure by fostering doubts about whether the exposure is being conducted correctly. However, levels of perfectionism have not been associated with response to OCD treatment [41].

While these non-responders to B4DT benefited from the structured, therapist-guided format, they may not have fully experienced themselves as autonomous agents capable of sustaining change independently. Our data conceptualized non-response not as a complete absence of change, but as difficulty transferring and sustaining treatment-related changes from the supported treatment setting to everyday life. From the perspective of the B4DT framework, this transfer difficulty may reflect incomplete consolidation of the intended shift toward approaching anxiety in the service of life beyond OCD. Within this framework, treatment success involves not only symptom improvement but also change in how patients relate to feared situations. Because intention was not directly assessed, this remains a theory-informed interpretation.

### 4.1. Implications for Treatment Optimization

The most direct clinical implication of the interviews concerns the transition to independent practice. Hence, a more systematic progression from guided to self-guided exposure within the treatment days may help patients experience themselves as independent agents of change and may support generalization beyond the treatment context. Furthermore, optional booster sessions or brief therapist contacts during the first weeks after treatment may be useful, but these should be designed to support autonomy rather than provide reassurance. Additionally, treatment optimization may require focusing on strengthening the transition from therapist-supported exposure to autonomous, self-guided, life-oriented action. The rationale for intention-based exposure may be emphasized more strongly. This may help distinguish active commitment to change from compliance with therapist instructions or courageous “pushing through” feared situations. Lastly, addressing societal and professional myths about the need for therapy to be long-term may be a key step toward improving engagement and long-term outcomes.

### 4.2. Limitations

Despite the insights gained, this study has several limitations. First, the analysis was based on qualitative interviews with a small sample, limiting the generalizability of the findings. In relation to this, because the interviews did not directly assess participants’ intentions during exposure, the construct of an ‘intention shift’ was interpreted through the B4DT framework rather than derived directly from participants’ own terminology. As a result, the study cannot determine whether an incomplete intention shift constituted a mechanism underlying non-response. Additionally, from the already small pool of 12 non-responders, only eight have participated in the interviews. Hence, the findings apply primarily to this specific subgroup and require replication in larger and more diverse samples. Future research should include larger and more diverse samples to enhance the representativeness of the results and employ quantitative methods to further substantiate the hypotheses generated from this study. Second, the extent to which retrospective bias may have influenced participants’ accounts remains uncertain. Evaluations of therapeutic experiences are shaped by multiple factors, including prior treatment history, current symptomatology, and subjective expectations [44,45]. Third, the study relied exclusively on participants’ self-reports and did not incorporate objective measures of treatment outcome. In addition, only a subset (eight out of 12) of the identified non-responders participated in the interviews, which may have introduced selection bias [46]. Finally, the influence of individual characteristics, such as personality traits or comorbid disorders, was not systematically investigated.

### 4.3. Future Research

Future studies should explore how the factors discussed here may moderate treatment engagement and outcomes to provide a more nuanced understanding of B4DT efficacy. Specifically, research should examine when symptom deterioration occurs, whether more intensive therapist support improves outcomes, and how much time patients spend practicing independently during the four treatment days, including potential reasons for limited independent practice. Further, there should be a measure for the shift in intention. Comparisons with responders are also needed to clarify whether successful outcomes are associated with higher levels of motivation, courage, or engagement. Moreover, research should distinguish between patients who do not improve during treatment and those who initially improve but are unable to maintain their gains. Future trials could compare standard B4DT with modified versions incorporating early booster sessions and agency-building either before or during B4DT. Such research may clarify how concentrated interventions can best balance therapist support with the cultivation of independence.

## 5. Conclusions

Although the participants in this study were quantitatively classified as non-responders, they nevertheless described meaningful psychological benefits from the B4DT. Rather than reflecting an absence of change, non-response may therefore indicate that the intended shift from symptom control to approaching anxiety in service of personally meaningful action was not fully consolidated. Strengthening this intention shift, while explicitly supporting patients’ ability to act on it independently in everyday life, may enhance the treatment effects of B4DT.

## Figures and Tables

**Table 1:** Baseline Demographics and Psychopathology of Responders vs. Non-Responders.

Variable	Responders *n* = 20	Non-Responders *n* = 12	Statistics
Sociodemographic Characteristics			
Gender, *n* (%)			Fisher’s Exact *p* = 1.00, *OR* = 1.07, 95% CI [0.25, 4.59]
Female	12 (60)	7 (58.3)	
Male	8 (40)	5 (41.7)	
Age in years, *M* (*SD*)	31.40 (10.65)	42.67 (14.20)	*t* (30) = 2.556, *p* = 0.016 *d* = 0.93, 95% CI [0.17, 1.68]
Occupation, *n* (%)			Fisher’s Exact *p* = 0.096 Cramér’s *V* = 0.5
Full-time	7 (35)	4 (33.3)	
Part-time	2 (10)	3 (25)	
Student/in training	7 (35)	0	
Retired	0	1 (8.3)	
Unemployed/other	4 (20)	4 (33.3)	
Relationship status, *n* (%)			Fisher’s Exact *p* = 0.14 Cramér’s *V* = 0.38
Not partnered	10 (50)	3 (25)	
Partnered/married	10 (50)	9 (75)	
Illness duration in years, *M* (*SD*)	15 (12.38)	23 (17.55)	*t* (29) = 1.626, *p* = 0.12 *d* = 0.60, 95% CI [−0.14, 1.33]
Age at OCD onset, *M* (*SD*)	17 (6.68)	19 (8.90)	*t* (29) = 0.765 *p* = 0.45 *d* = 0.28, 95% CI [−0.45, 1.00]
Medication, *n* (%)			
Antidepressant	0	1 (8.3)	Fisher’s Exact *p* = 0.38 *OR* = 5.3, 95% CI [0.20, 142.28]
Benzodiazepine	0	0	-
Comorbidities, *n* (%)			
Major depression, current episode	4 (20)	2 (16.7)	Fisher’s Exact *p* = 1.00 *OR* = 0.8, 95% CI [0.12, 5.2]
Major depression, recurrent, without current episode	6 (30)	4 (33.3)	Fisher’s Exact *p* = 1.00 *OR* = 1.17, 95% CI [0.25, 5.41]
Eating disorder (current)	1 (5)	1 (8.3)	Fisher’s Exact *p* = 1.00 *OR* = 1.73, 95% CI [0.10, 30.45]
Panic disorder (lifetime)	3 (15)	1 (8.3)	Fisher’s Exact *p* = 1.00 *OR* = 0.52, 95% CI [0.05, 5.60]
Social phobia	3 (15)	0	Fisher’s Exact *p* = 0.27 *OR* = 0.20, 95% CI [0.01, 4.23]
Generalized anxiety disorder	2 (10)	0	Fisher’s Exact *p* = 0.52 *OR* = 0.30, 95% CI [0.01, 6.70]
Psychopathology			
Y-BOCS total, *M* (*SD*)	25 (3.17)	26 (5.26)	*t* (30) = 0.48, *p* = 0.63; *d* = 0.18, 95% CI [−0.54, 0.89]
Y-BOCS obsessions, *M* (*SD*)	12 (1.99)	13 (2.90)	*t* (30) = 0.25, *p* = 0.80 *d* = 0.09, 95% CI [−0.63, 0.81]
Y-BOCS compulsions, *M* (*SD*)	13 (1.60)	13 (2.52)	*t* (30) = 0.69, *p* = 0.50 *d* = 0.25, 95% CI [−0.47, 0.97]
PHQ-9, *M* (*SD*)	10 (5.47)	10 (5.52)	*t* (30) = −0.09, *p* = 0.93 *d* = 0.03, 95% CI [−0.75, 0.68]
OCI-R washing, *n* (%)	13 (65)	6 (50)	Fisher’s Exact *p* = 0.47, *OR* = 0.54, 95% CI [0.13, 2.31]
OCI-R checking, *n* (%)	14 (70)	10 (83.3)	Fisher’s Exact *p* = 0.68, *OR* = 2.14, 95% CI [0.36, 12.90]
OCI-R symmetry, *n* (%)	6 (30)	4 (33.3)	Fisher’s Exact *p* = 1.0, *OR* =1.17, 95% CI [2.5, 5.41]
OCI-R neutralizing, *n* (%)	5 (25)	2 (16.7)	Fisher’s Exact *p* = 0.68, *OR* = 0.60, 95% CI [1.0, 3.72]
OCI-R obsessing, *n* (%)	18 (90)	11 (91.7)	Fisher’s Exact *p* = 1.0, *OR* = 1.2, 95% CI [0.10, 15.11]
OCI-R hoarding, *n* (%)	5 (25)	1 (8.3)	Fisher’s Exact *p* = 0.37, *OR* = 0.27, 95% CI [0.03, 2.68]

Note. Y-BOCS = Yale-Brown Obsessive Compulsive Scale; PHQ-9 = Patient Health Questionnaire-9; OCI-R = Obsessive Compulsive Inventory–Revised. OCI-R symptom dimensions were classified using a cutoff of ≥4 on the OCI-R subscales. Exact tests were used due to small expected cell counts: Fisher’s exact test for 2 × 2 tables. Effect sizes are reported as Hedges’ g for continuous variables, odds ratios for dichotomous variables, and Cramér’s V for categorical variables with more than two levels. The table includes all 12 non-responders, not just the eight non-responders who were interviewed. All variables included in the analyses were measured at baseline, one day before the B4DT.

**Table 2:** Demographics and Psychopathology of the Eight Interviewed Non-Responders.

Participant	Gender	Age	Y-BOCS T0	Y-BOCS T1	Y-BOCS T2	Comorbidities	Time from B4DT to Interview (Months)
PM-01	Female	33	20	14	18	Major depressive disorder, recurrent, without current episode	20
PM-02	Male	26	22	19	17	Major depressive disorder, recurrent, without current episode	20
PM-03	Male	36	18	15	17	Major depressive disorder, recurrent, without current episode; panic disorder, lifetime	17
PM-04	Female	67	26	22	20	None	14
PM-05	Female	61	28	25	24	Major depressive episode, current	14
PM-06	Female	28	26	15	27	Major depressive episode, current	10
PM-07	Male	62	32	22	29	Eating disorder, current	11
PM-08	Female	45	24	13	17	Major depressive disorder, recurrent, without current episode	11

Note. Y-BOCS = Yale-Brown Obsessive Compulsive Scale, total score. T0 refers to baseline, T1 refers to post-treatment, T2 refers to follow-up 3 months after treatment. Age and comorbidities were assessed at T0.

**Table 3:** Therapists’ Ratings of Participants’ Efforts Throughout B4DT Treatment Days.

Patient	Day	Completed Exposures as Planned ^2^	Spontaneously Used Opportunities for Exposures ^2^	Managed Exposure by Applying LET Technique ^2^	Avoidance During Exposure ^1^	Overall Effort (1–6) ^3^
P-01	2	100	100	100	0	6
	3	80	100	100	0	6
P-02	2	100	90	90	20	6
	3	70	100	80	20	6
P-03	2	70	100	70	80	4
	3	90	90	80	20	5
P-04	2	70	10	40	60	3
	3	100	100	100	20	5
P-05	2	100	100	90	10	6
	3	100	70	70	10	5
P-06	2	90	70	80	10	5
	3	90	90	90	20	6
P-07	2	90	100	90	30	6
	3	90	60	80	10	6
P-08	2	90	100	90	30	6
	3	90	80	90	20	6

Note. Participants were rated regarding their effort on days 2 and 3 of the treatment, including their performance in the afternoon/evening after the official treatment. ^1^ The degree to which participants held back or applied safety behavior, compulsions, or avoidance during ERP. ^2^ Questions were rated on a scale from 0 to 100. ^3^ The overall effort was rated on a scale from 1 to 6, with 6 being the highest score.

**Table 4:** Overview of Final Categories and Subcategories Derived From the Qualitative Analysis.

Main Category	Subcategory	Description	Illustrative Quotation
Initial Engagement	Hope	Participant entered B4DT with hope that the treatment could lead to meaningful improvement.	“I have had this disorder for more than 50 years and I was looking for something new and I found this, so I signed up in the hope that it might help.” [P-07]
	Positive expectations	Participant anticipated that the intensive treatment format would substantially reduce their OCD symptoms and viewed B4DT as a promising treatment opportunity.	“Before the week started, my expectation was that my OCD would improve significantly. And I have to say I entered the week fully motivated.” [P-06]
Positive Change During Treatment	Courage through exposure	Engaging in challenging exposure exercises increased participants’ sense of courage.	“I have become more courageous, exposing myself to situations that I would otherwise never have considered. I have learned that difficult situations are bearable and not as bad as I thought they would be.” [P-03]
	Understanding OCD and B4DT	Participant developed a clearer understanding of OCD, the role of compulsions and avoidance, and the rationale underlying B4DT.	“Receiving such a detailed explanation of the theoretical background was important and helpful.” [P-07]
	Peer support	The group format made participants feel validated, less isolated, and motivated them to engage during treatment.	“Getting through the week as a group and sharing experiences with others really helped me a lot.” [P-06]
Transition to Independent Practice	Late symptom recognition	Participant discovered new OCD symptoms within the treatment, leaving limited time to practice suitable exposure exercises.	“I think it was missing a bit more of a focus on the fact that it’s really in my head, but I only became aware of everything in the course of the week.” [P-06]
	Safety behavior during exposure	Participant experienced the therapist’s presence as providing reassurance, external permission, or a sense of safety during exposure, potentially using these as safety cues.	“I didn’t [feel afraid]. The therapist actually gave me safety, so it wasn’t actually painful.” [P-05]
	Desire for ongoing support	Participant expressed a wish for continued therapeutic guidance, follow-up contacts, or booster sessions after the concentrated treatment days.	“During the four days, it was very motivating, but I noticed that afterwards I missed having someone by my side.” [P-01]
	Treatment time	Participants perceived the available time for guided exposure, practice, and preparation for independent implementation as insufficient.	“You can’t do that in such a short time. I didn’t have enough time; maybe three more days would have been enough.” [P-05]
Amplifiers	Limited social support	A lack of encouragement or practical support from significant others made it more difficult to continue applying treatment principles in everyday life.	“My relationship at the time—I didn’t receive any support. It actually drained my energy because I was trying to keep the relationship going.” [P-03]
	Occupational stress	Work-related demands and stress competed with participants’ capacity to maintain self-guided exposure and behavioral change.	“I find it difficult to set boundaries, and I think that during those days, I did not direct all my energy toward the treatment because I was also focused on my work.” [P-08]
	Diagnostic uncertainty	Uncertainty regarding the OCD diagnosis or perceived treatment fit reduced participants’ sense of preparedness or confidence in the treatment approach.	“The prolonged uncertainty during the outpatient assessment reinforced my feeling that I was not the right target group and left me feeling insecure. Sometimes I wonder whether it was OCD at all.” [P-02]
	Depressive symptoms	Depressive symptoms reduced participants’ energy and capacity to sustain exposure practice after treatment.	“After about a month, I was no longer able to maintain what I had learned during the week—the depressive symptoms came back and made everything harder.” [P-06]
	Perfectionism	Concerns about performing exposure correctly and fears of making mistakes made independent practice feel more difficult or overwhelming.	“This idea that I mustn’t do anything wrong, this absolute perfectionism. That made it difficult for me.” [P-04]

Note. The table summarizes the categories and subcategories derived from the qualitative analysis. Quotations were selected to illustrate participants’ accounts and were lightly shortened where necessary for clarity.

## Data Availability

Data can be made available upon request.

## Abbreviations

The following abbreviations are used in this manuscript:

B4DT	Bergen 4-Day Treatment
cET	Concentrated Exposure Therapy
ERP	Exposure with Response Prevention
LET	Leaning into Anxiety
MINI	MINI International Neuropsychiatric Interview
OCD	Obsessive–Compulsive Disorder
YBOCS	Yale-Brown Obsessive Compulsive Scale

## Appendix A

Interview: Evaluation of the B4DT for Obsessive-Compulsive Disorder from the Perspective of Patients
Thank you very much for taking the time today to answer a few questions about the B4DT. Your feedback will help us to better understand which aspects of the treatment were helpful for patients and which areas we may still be able to improve. We are particularly interested in whether anything was missing from your perspective and which procedures could be further optimised.
The aim of this interview is therefore for you to describe your experiences with the B4DT in your own words.
The interview will take approximately 45 min. First, I will ask you a few questions about your current well-being. In the second part of the interview, we will focus on your experiences during the intensive treatment programme, including what was helpful for you and what you may have found less helpful or less satisfactory. Please provide as much detail as possible in response to the aspects I ask you about.
As I have already explained to you, the interview will be audio-recorded with your consent. The data will be pseudonymised for analysis, and the audio recording will be deleted once the interview has been transcribed.
Do you have any questions before we begin?

1. General Questions

[approx. 5 min]

*For patients you do not know, begin with introductory questions such as: “When did you take part?”* etc.

1a. How are you feeling at the moment?

1b. What led you to contact us at the time? What were your expectations of the treatment?

2. Changes

[approx. 10 min]

Using the following questions, I would like to find out whether you noticed any changes in yourself during or after the B4DTand, if so, what kind of changes these were. We will then look at how you evaluate these changes.

2a. Have you noticed any changes in yourself since completing the intensive treatment programme?

2b. If so, what changes have you noticed?

Follow-up questions:

- Have you noticed any changes in your thoughts, behaviour, or emotions?
- What specific ideas, insights, or strategies have you taken away from the intensive treatment programme, including in relation to yourself or other people?
- Have other people drawn your attention to any changes they noticed in you?

2c. Has anything changed for the worse for you since completing the intensive treatment programme?

2d. Is there anything you had wanted to change that has not changed since the beginning of the intensive treatment programme?

3. Evaluation of Changes

[approx. 10 min]

*Go through each previously noted change and ask the participant to rate it on the following three scales.*

3a. Expectedness of the Change

Was this change rather surprising to you, or had you expected it in this or a similar form?

How was it regarding the change “___”?

1. I very much expected this change.
2. I somewhat expected this change.
3. I neither expected this change nor was I surprised by it.
4. I was somewhat surprised by this change.
5. I was very surprised by this change.

3b. Likelihood of the Change Without Treatment

How likely do you think it is that this change would have occurred even without the B4DT? Please rate each change in terms of how likely it would have been if you had not taken part in therapy.

1. Very unlikely without therapy—it clearly would not have occurred.
2. Rather unlikely without therapy—it probably would not have occurred.
3. Neither likely nor unlikely—it is not possible to say.
4. Rather likely without therapy—it probably would have occurred.
5. Very likely without therapy—it definitely would have occurred.

3c. Personal Importance of the Change

How important or meaningful is this change to you personally?

1. Not at all important
2. Slightly important
3. Moderately important
4. Very important
5. Extremely important

4. Attributions

[approx. 5 min]

What do you think caused the different changes you have described? In other words, what do you think may have brought about these changes?

This may include factors both outside the B4DT and within the B4DT itself.

5. Problematic Aspects

[approx. 5 min]

*Offer prompts if the patient cannot think of anything.*

5a. Which aspects of the B4DT did you experience as rather obstructive, unhelpful, negative, or disappointing? This may include general aspects or specific events.

5b. Were there any elements of the B4DT that were difficult or distressing, but nevertheless acceptable or perhaps even helpful? Which elements were these? Please provide examples, such as general aspects or specific events.

5c. Was there anything missing from the intensive treatment programme? What would have made the programme more effective or more helpful?

6. Resources

[approx. 5 min]

6a. Which personal strengths do you think helped you to make use of the content or techniques of the B4DT to manage your difficulties? This may include things you are good at or personal characteristics.

6b. Which aspects of your life situation during the treatment helped you to make use of the content or techniques of the B4DT to manage your difficulties? This may include family, work, relationships, or living situation.

7. Limitations and Difficulties

[approx. 5 min]

7a. In your view, which personal factors made it more difficult for you to address your difficulties in treatment? By this, I mean factors related to you as a person.

7b. Which aspects of your life situation at the time of treatment made it more difficult to make use of therapy in order to manage your difficulties? This may include family, work, relationships, or general life circumstances.

8. Procedure of the Intensive Treatment Programme

[approx. 10 min]

*Offer prompts if no feedback is provided.*

8a. How did you experience the preliminary sessions? Looking back, was there anything missing in terms of preparation? What would you have liked to know in advance?

8b. How would you summarise the overall procedure of the intensive treatment programme, including the preliminary sessions, assessments, the intensive treatment itself, interviews, and the booster session, if applicable?

8c. Which aspects related to the study were obstructive, unhelpful, negative, or interfered with the intensive treatment programme? Please provide examples.

9. Suggestions and Closing Questions

[approx. 5 min]

Do you have any suggestions for us regarding the intensive treatment programme?

Is there anything else you would like to tell me?

Is there anything you would have liked me to ask you?

Or was there anything you were concerned I might ask you?

Thank you very much for taking the time to answer all these questions. Your feedback is very valuable to us. We hope to continuously adapt and further optimise the B4DTbased on your experiences, so that many more patients may have the opportunity to benefit from it in the future.
